# Primary care influenza‐like illness surveillance in Ho Chi Minh City, Vietnam 2013‐2015

**DOI:** 10.1111/irv.12574

**Published:** 2018-07-07

**Authors:** Stacy Todd, Nguyen Thi Cam Huong, Nguyen Thi Le Thanh, Nguyen Ha Thao Vy, Nguyen Thanh Hung, Tran Thi Nhu Thao, Huynh Thi Phuong, Rogier van Doorn, Vu Thi Ty Hang, Nguyen Van Vinh Chau, Jonathan M. Read, David G. Lalloo, Maciej F. Boni

**Affiliations:** ^1^ Liverpool School of Tropical Medicine Liverpool UK; ^2^ Tropical and Infectious Disease Unit Royal Liverpool and Broadgreen University Hospital Liverpool UK; ^3^ Oxford University Clinical Research Unit Wellcome Trust Major Overseas Programme Ho Chi Minh City Vietnam; ^4^ Hospital for Tropical Diseases Ho Chi Minh City Vietnam; ^5^ Centre for Tropical Medicine and Global Health Nuffield Department of Medicine University of Oxford Oxford UK; ^6^ Centre for Health Informatics Computing and Statistics Lancaster Medical School Lancaster University Lancaster UK; ^7^ Institute of Infection and Global Health University of Liverpool Liverpool UK; ^8^ Center for Infectious Disease Dynamics Department of Biology Pennsylvania State University University Park PA USA

**Keywords:** epidemiology, human, influenza, primary health care, Vietnam

## Abstract

**Background:**

Year‐round transmission of influenza has been detected in Vietnam through both national surveillance and other epidemiological studies. Understanding the demographic and clinical features of influenza‐like illness (ILI) presenting to primary care in urban Vietnam is vital to understand these transmission dynamics.

**Methods:**

An observational study of patients with ILI in Ho Chi Minh City, Vietnam, was conducted between August 2013 and November 2015 in a mix of public and private primary care settings. Molecular testing for influenza A and influenza B and 12 other respiratory viruses was performed.

**Results:**

A total of 1152 ILI patients were recruited. 322 and 136 subjects tested positive for influenza A and influenza B, respectively. 193 subjects tested positive for another respiratory virus; most commonly rhinovirus and parainfluenza virus 3. Influenza was detected in 81% of the 116 study weeks. Three peaks of influenza activity were detected; an H3N2 peak April‐June 2014, an influenza B peak July‐December 2014, and a mixed H3N2 and H1N1 peak March‐September 2015. Subjects recruited from private clinics were more likely to have higher income and to have reported previous influenza vaccination. Antibiotic use was common (50.3%) despite limited evidence of bacterial infection.

**Conclusion:**

Influenza in southern Vietnam has complex transmission dynamics including periods of intense influenza activity of alternating types and subtypes. Broadening surveillance from hospital to the community in tropical settings is feasible and a valuable for improving our understanding of the global spread and evolution of the virus.

## 1 BACKGROUND

Influenza viruses circulate globally and seasonal epidemics are thought to be associated with 3‐5 million severe clinical infections and 250 000 to 500 000 deaths each year.^1^, ^2^ Morbidity and mortality are highest at the extremes of age but individuals of all ages are affected with repeat infections throughout their lifetime. In temperate countries, predictable winter epidemics occur, but transmission dynamics in tropical settings are more irregular. For many decades, influenza was thought not to be a disease of importance in tropical countries. This has changed considerably in the last 10 years.^3^ Research efforts are ongoing to increase knowledge and understanding of how tropical transmission influences global influenza transmission. Vietnam, as one of the most populous countries in the tropics, is a potentially globally important site for influenza dynamics, both for its possible contribution to global virus evolution^4^, ^5^ and as a high‐risk environment for the emergence of potentially pandemic strains such as highly pathogenic avian influenza subtype H5N1.

Year‐round transmission of influenza has been detected in Vietnam through both national surveillance^6^, ^7^ and phylogenetic analysis.^8^ Simultaneous circulation of multiple influenza types and subtypes has been documented^6^, ^8^, ^9^, ^10^, ^11^ along with patients having more than one influenza infection in one season.^9^ Approximately 15%‐20% of patients presenting to hospitals with influenza‐like illness (ILI) have virologically confirmed influenza,^6^, ^7^ and influenza is associated with up to 14% of community‐acquired pneumonia presenting to hospitals.^12^ However, complementary data on community ILI incidence are limited. As national surveillance programmes 6, 7 have focused largely on hospital sites, estimates from these studies are likely to underestimate true clinical burden and attack rates in the community. Seasonal influenza vaccine is not currently provided through public health services in Vietnam and there is minimal, although increasing, use in the private sector.^13^ The optimal vaccination strategy for seasonal influenza in Vietnam is still to be established.^14^

The objectives of this study were to describe the dynamics and demographic/clinical characteristics of ILI presenting to primary care services within an urban setting in Vietnam over a 27‐month period and to estimate the proportion of ILI caused by influenza A and influenza B.

## 2 METHODS

### 2.1 Summary of design and conduct of the observational study

This observational study was conducted between August 2013 and November 2015 at outpatient clinics and community medical practitioners in Ho Chi Minh City (HCMC), Vietnam (private clinics were drawn from the an ongoing mHealth surveillance study 11). These clinics were considered representative of primary care within Vietnam.^15^ Individuals were invited to join the study if they were between 10 and 70 years of age, had symptoms for <72 hours and met the ECDC definition of influenza‐like illness.^16^ One anterior nasal swab and one throat swab were collected at recruitment and transported in a single tube of viral transport medium to a central laboratory before being stored at −20°C within 24 hours.

Clinical characteristics of the initial ILI episode were reported by subjects to study staff. No inpatient data were collected in the event of hospitalisation. No additional objective information was available from the subjects’ usual treating clinician.

This study was approved by the Scientific and Ethical Committee of the Hospital for Tropical Diseases, Ho Chi Minh City, Vietnam and by the Liverpool School of Tropical Medicine Research Ethics Committee, UK. Letters of agreement supporting the involvement of the community medical clinics were obtained from the Ho Chi Minh City Department of Health.

### 2.2 Sample analysis

Samples were stored at −20°C within 24 hours of collection and then stored at −80°C after initial PCR testing. Respiratory samples were batch tested monthly for influenza A and influenza B using standard polymerase chain reaction (PCR) techniques.^17^ Samples positive for influenza A were then subtyped as H3N2 or H1N1. Testing for twelve other respiratory viruses was performed on samples which tested negative for influenza using an in‐house multiplex PCR assay which has been described previously.^18^ The viruses included were adenovirus, bocavirus, coronavirus 1/2, enterovirus, metapneumovirus, parainfluenza viruses 1‐4, rhinovirus, respiratory syncytial virus A and B.

### 2.3 Statistical analyses

The primary outcome was PCR‐confirmed influenza A in nasal/throat swabs. This included both mono‐infections with influenza A infections and co‐infections with influenza B.

Continuous variables that were normally distributed were compared with *t* test or ANOVA as appropriate. Tukey's HSD was used for post hoc testing following ANOVA where appropriate. Continuous variables that were non‐normally distributed were compared using Mann‐Whitney or Kruskal‐Wallis tests, depending on the number of groups. Categorical variables were compared using Fisher's exact test, Mann‐Whitney or chi‐squared tests, as appropriate.

Analysis of overall prevalence of influenza as a cause of ILI was performed on the total study population; weekly prevalence was also calculated. Weekly influenza transmission intensity was categorised by percentage of ILI subjects testing influenza‐positive using WHO thresholds^19^ as zero (0%), low (1%‐10%), medium (11%‐20%), high (21%‐30%) and very high (>30%). A peak of influenza activity was defined as four or more consecutive weeks where influenza transmission intensity was high or very high. Weeks when no recruitment was performed because of clinic closures were excluded from analysis; weeks during which clinics were open but no patients were recruited were included in the analysis. Interactions between influenza and non‐influenza respiratory virus circulation were investigated using Pearson's correlation and cross‐correlation with weekly time lags.

Demographic and clinical characteristics were compared between predefined groups based on influenza infection status. The initial analysis was planned to be performed between three groups: influenza A positive, influenza B positive and non‐influenza ILI with a subsequent analysis between influenza A subtypes (H1N1 and H3N2) if numbers were sufficient. Where appropriate, analysis was stratified by age. Age was preferentially used as a continuous variable, otherwise age was categorised as per recommendations from the Consortium for the Standardisation of Influenza Seroepidemiology (CONSISE, 5‐9, 10‐19, 20‐44, 45‐65, 65+).^20^ Comparison between subjects recruited at public and private clinics was also performed. Logistic regression was used to investigate the effect of (1) household age structure (total number of household contacts, total number of household contacts in each CONSISE age class) and (2) recent household ILI on the risk of any influenza (influenza A or influenza B) compared to non‐influenza ILI.

Age, gender and household size data were compared to national Vietnamese Census Data^21^ according to influenza infection status and for all study subjects combined. Age and gender comparisons were also made with Ho Chi Minh City census data. Household size comparisons were made to Vietnamese National Urban Average census data. For each of the infection status groups, the expected proportion was the census point estimate; 95% binomial confidence intervals of the expected proportion were calculated using the number of subjects in that category. Goodness of fit was tested (χ^2^) using the census proportions to determine whether study distributions were significantly different from the general Vietnamese population.

All statistical analysis was performed using R Statistical Software v3.3.2.^22^ Packages used for analysis were ggplot2, gamm4, mgcv, Epi, survival and MASS.

## 3 RESULTS

A total of 1152 subjects with influenza‐like illness were recruited between 8 August 2013 and 27 November 2015 (121 weeks). The majority of patients were recruited from hospital outpatient settings (836/1152, 72.6%).

### 3.1 ILI dynamics

**Figure 1:**
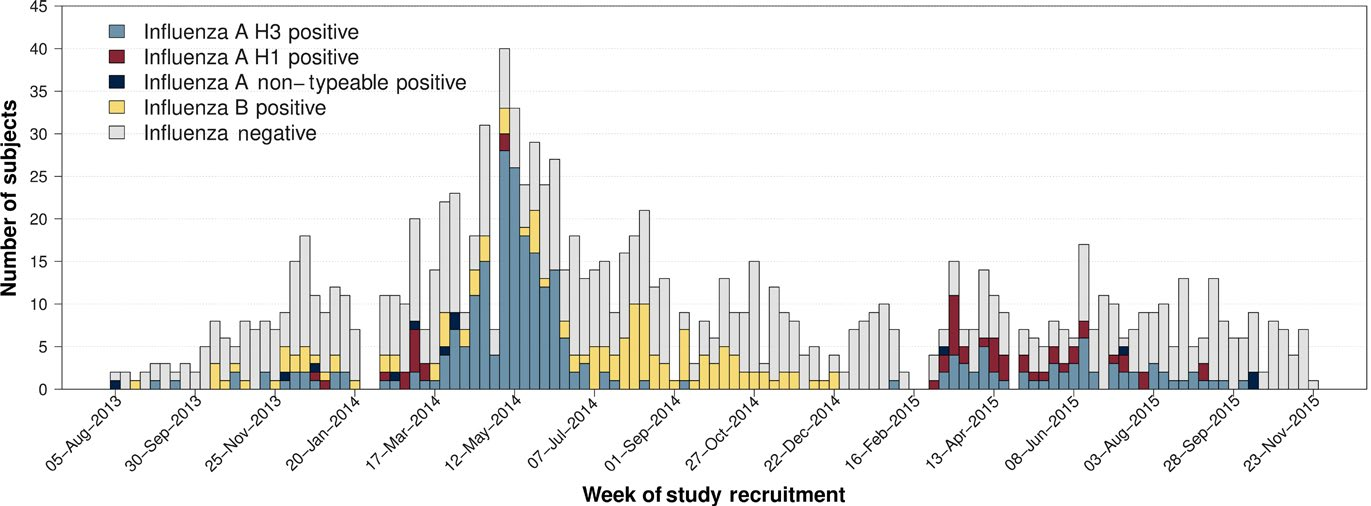
Weekly study recruitment timeline by influenza PCR result. Study clinics were closed to recruitment during national public holidays corresponding to study weeks 26, 27, 81, 82 and 91

**Figure 2:**
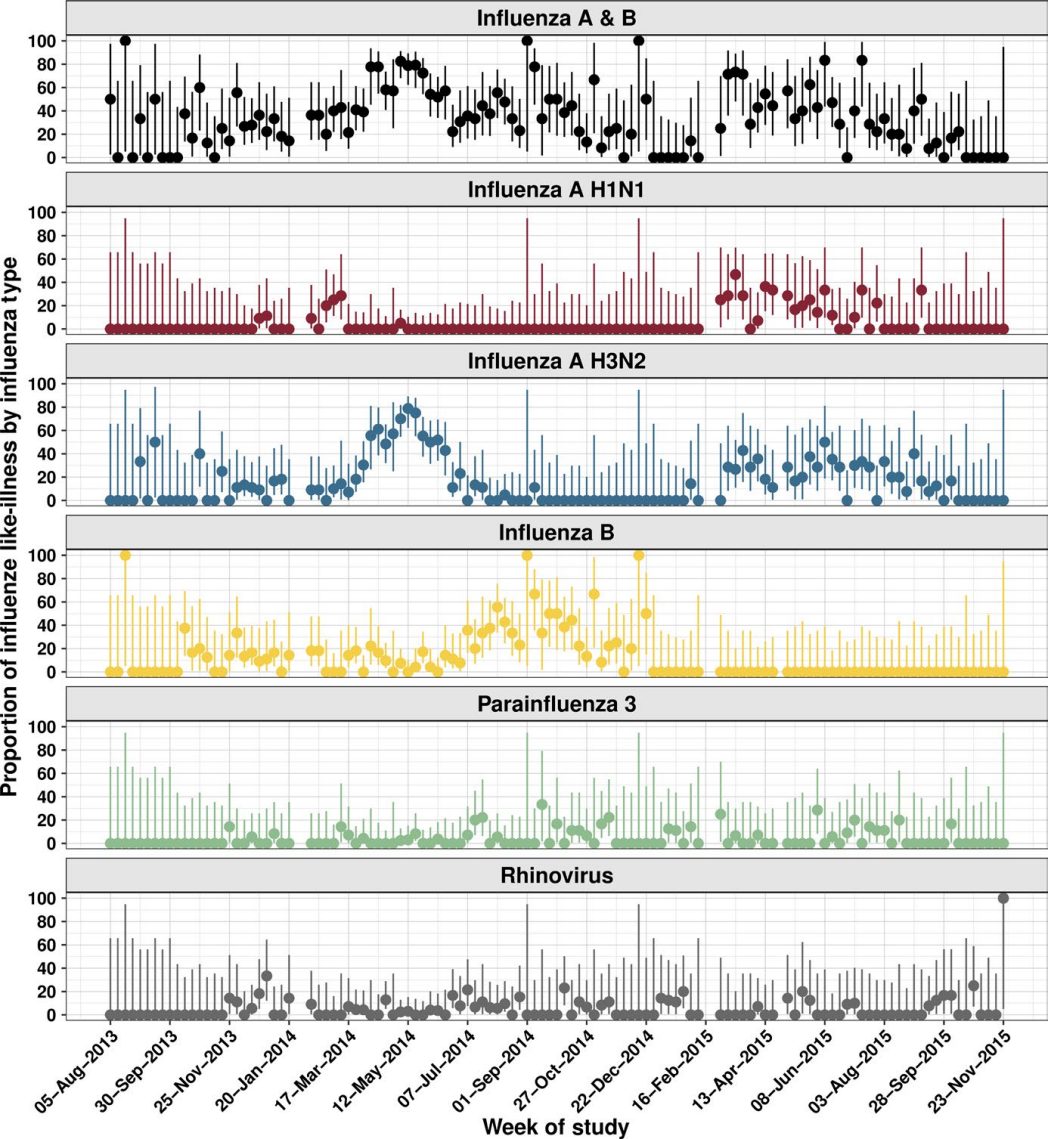
Proportion of ILI caused by PCR‐confirmed influenza with Binomial 95% confidence intervals

About 56.5% of subjects had at least one virus detected by PCR (n = 651/1152; Table S1). 40.6% of ILI cases had PCR‐confirmed influenza (n = 458/1152; Figure 1). Influenza A was detected in 322 subjects (27.9%) and influenza B in 136 subjects (11.8%). H3N2 was the commonest circulating influenza A subtype (80.1%, n = 258/322). H1N1 was detected in 52 subjects (16.1%), and 3.7% of influenza A samples could not be subtyped. Recruitment was not performed in the 5 weeks when clinics were closed because of public holidays (study weeks 26, 27, 81, 82 and 91). Influenza was detected in most weeks when study recruitment was performed (81.0%, n = 94/116). Influenza A was detected in 68 study weeks (58.6%, n = 68/116), with H3N2 present more frequently than H1N1 (62 vs 25 weeks, 53.4% vs 21.5%). Influenza B was detected in 51 of 116 study weeks (43.9%). Three periods of sustained high or very high transmission were identified (Figure 2). An H3N2 peak occurred between 31 March 2014 and 30 June 2014 (study weeks 35‐48) which was immediately followed by an influenza B peak between 07 July 2014 and 22 December 2014 (study weeks 49‐73). Finally, a mixed H1N1 and H3N2 peak in activity was detected between 02 March 2015 and 07 September 2015 (study weeks 83‐110).

Of samples which tested negative for influenza, 27.8% (n = 193/694) tested positive for another respiratory virus (Table S1 and Figure S1). Rhinovirus and parainfluenza virus 3 (PIV3) were the most commonly detected viruses at 8.8% (n = 61/694) and 6.3% (n = 44/694), respectively. Multiple viruses were detected in 2.3% of cases. Rhinovirus was detected in 45 study weeks (38.8%, n = 45/116) and PIV3 in 36 weeks (31%, n = 36/116; Figure 2). H3N2 circulation was correlated with Adenovirus (ρ = .24 (95% CI: 0.07‐0.40)) and PIV3 (ρ = .19; 95% CI: 0.009‐0.35) activity; rhinovirus was significantly correlated with influenza B circulation (ρ = .33 [95% CI: 0.17‐0.48]). No lagged correlations were identified through cross‐correlation analysis. Results are presented without corrections for multiple comparisons, meaning that some of the more borderline trends could be spurious. (eg above uncorrected *P*‐value for H3N2‐PIV3 correlation is *P *=* *.04).

### 3.2 Age distribution

**Figure 3:**
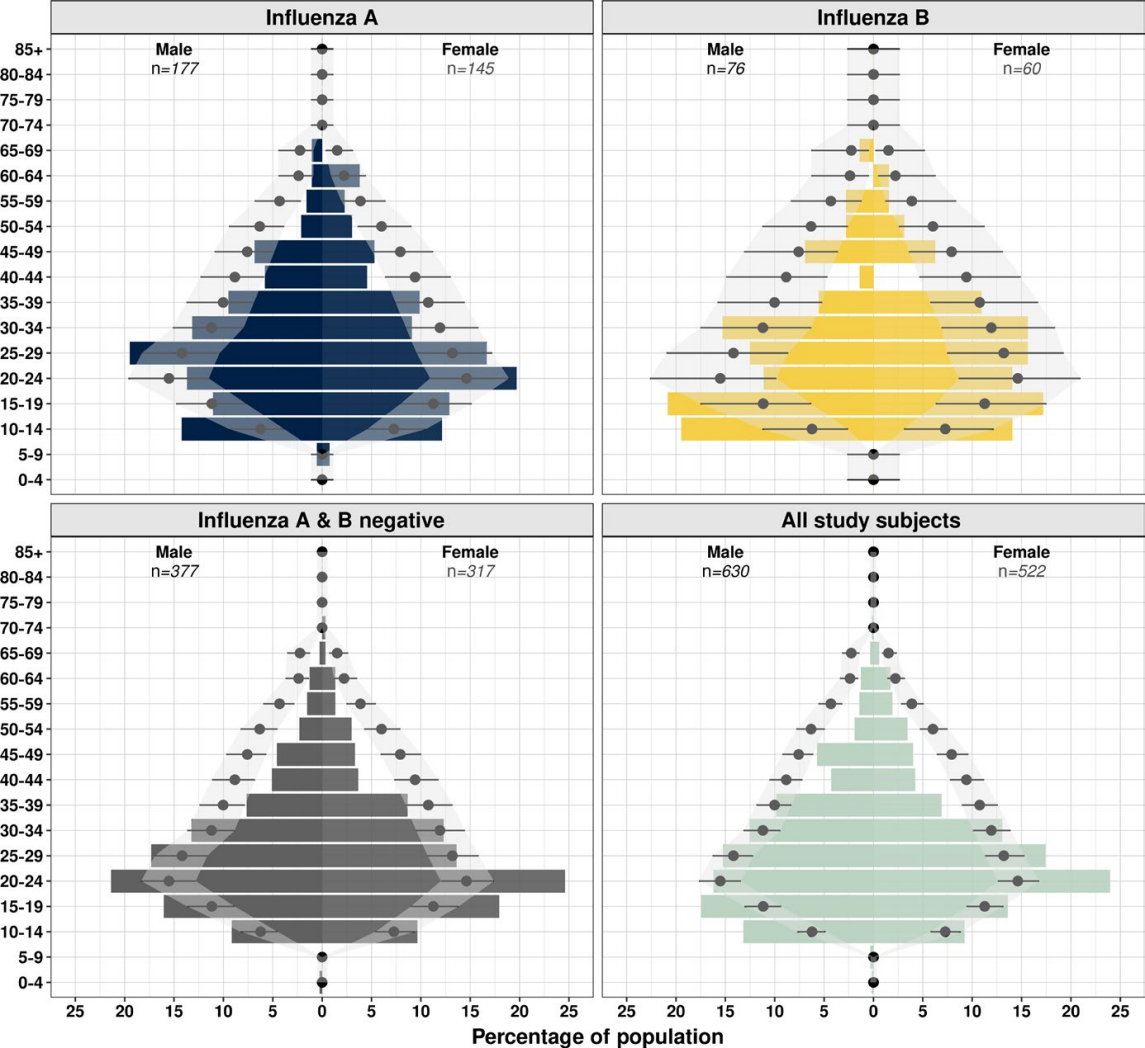
Age and sex distribution by infecting influenza type. Point and lines represent expected percentage for HCMC with binomial 95% CI. Coloured bars show percentages observed in this study. Expected values were limited to age groups included in the study protocol (10‐70 inclusive)

Age of recruited study participants was right skewed with a median age of 25.8 (IQR 19.2‐34.4). Median age of study participants did not vary by gender (Mann‐Whitney, *P*‐value .206) or influenza infection status (Kruskal‐Wallis, *P*‐value = .346). With the exception of subjects infected with influenza B, younger individuals were overrepresented in our study compared to the general population (Figure 3, Table S2). The comparison was the same using national Vietnamese or HCMC‐specific age distribution data (*t* test, all *P* values >.4).

### 3.3 Clinical characteristics

#### 3.3.1 Past medical history

At baseline, there was no significant difference in relevant past medical history (PMH) of individuals presenting with PCR‐confirmed influenza A, influenza B or non‐influenza ILI (Table S3). Chronic respiratory disease was uncommon in recruited subjects (2.4%, n = 23/953) but was reported more frequently in individuals aged 45 years or over (6.3% vs 1.8%, 6/95 vs 17/858; χ^2^ 5.106 *P* value .0238). Current smoking was reported in 14.9% of the study population, all of whom were male (n = 172/1152). About 36.1% of males over the age of 20 were current smokers (n = 156/432). 2.8% (n = 36/1152) of the study population reported an indication for influenza vaccination as defined by WHO; age > 65; PMH of COPD, congenital heart disease, heart failure, diabetes or asthma; healthcare worker. 2.2% of the study population reported receiving vaccination at some point in their life (n = 25/1152), but only one of these had a reported indication. All subjects who reported vaccination had received it after the emergence of the 2009 influenza pandemic.

#### 3.3.2 Clinical symptoms at presentation

Most subjects presented on the second or third day of symptoms (Table 1). Those with influenza B presented later than those with influenza A or those who were influenza negative (ANOVA *F*_2,1149_ = 15.07, *P *<* *.001); however, the difference in time to presentation was 0.28 (95% CI 0.14‐0.43) of a day (ie approx. 7 hours) which is not clinically important. All subjects had mild disease, with <1% of subjects reporting that they could not carry out their normal daily activities (n = 5/1152). Rhinorrhoea and cough were reported more frequently in those infected with influenza. Antibiotic use prior to enrolment in the study was common in all groups (53.0%, n = 611/1152) but was highest in those with influenza B (66.2%, n = 90/136; χ^2^ 11.6, *P* value = .003).

**Table 1:** Clinical symptoms at presentation

	Influenza A n (%)/med (IQR)	Influenza B n (%)/med (IQR)	Influenza negative n (%)/med (IQR)	χ^2^	*P* value
Days symptom onset	2 (2‐3)	3 (2‐3)	2 (2‐3)		
Normal tasks					
Yes	318 (98.8)	136 (100)	693 (99.9)	6.81	.0332
No	4 (1.2)	0 (0)	1 (0.1)		
Fever					
Yes	297 (92.2)	118 (86.8)	597 (86)	8.122	.0172
No	25 (7.8)	18 (13.2)	97 (14)		
Temp if known	38.5 (38‐39)	38.1 (38‐38.8)	38 (37.6‐38.7)		
Headache					
Yes	299 (92.9)	117 (86)	631 (90.9)	5.3837	.0678
No	23 (7.1)	19 (14)	63 (9.1)		
Rhinorrhoea					
Yes	278 (86.3)	110 (80.9)	484 (69.7)	35.1785	<.001
No	44 (13.7)	26 (19.1)	210 (30.3)		
Cough					
Yes	307 (95.3)	129 (94.9)	598 (86.2)	24.4923	<.001
No	15 (4.7)	7 (5.1)	96 (13.8)		
Sore throat					
Yes	287 (89.1)	120 (88.2)	599 (86.3)	1.6946	.4286
No	35 (10.9)	16 (11.8)	95 (13.7)		
Myalgia					
Yes	292 (90.7)	123 (90.4)	634 (91.4)	0.194	.9075
No	30 (9.3)	13 (9.6)	60 (8.6)		
GI symptoms					
Yes	22 (6.8)	13 (9.6)	58 (8.4)	1.1481	.5632
No	300 (93.2)	123 (90.4)	636 (91.6)		
Malaise					
Yes	313 (97.2)	129 (94.9)	670 (96.5)	1.5792	.454
No	9 (2.8)	7 (5.1)	24 (3.5)		
Paracetamol					
Yes	268 (83.2)	119 (87.5)	585 (84.3)	1.3312	.514
No	54 (16.8)	17 (12.5)	109 (15.7)		
Antiviral					
Yes	1 (0.3)	1 (0.7)	2 (0.3)	0.6743	.7138
No	321 (99.7)	135 (99.3)	692 (99.7)		
Antibacterial					
Yes	172 (53.4)	90 (66.2)	349 (50.3)	11.5506	.0031
No	150 (46.6)	46 (33.8)	345 (49.7)		
Vitamin					
Yes	124 (38.5)	67 (49.3)	277 (39.9)	4.9518	.0841
No	198 (61.5)	69 (50.7)	417 (60.1)		

### 3.4 Demographic characteristics

The commonest reported occupation was school pupil or student (26.7%, n = 308/1152) followed by manual labourers (21.4%, n = 247/1152) and shop assistants/traders (14.4%, n = 166/1152; Table S4). Subjects recruited from private clinics were significantly more likely to have professional jobs (28.7% (n = 90/316) vs 8.7% (n = 73/836), χ^2^ 72.1 (1), *P* value < .005). Contact with pigs was uncommon but contact with poultry was reported more frequently with 8.8% and 9.3% of subjects reporting weekly contact with live or dead poultry, respectively.

Median number of household members (including the study subject) was four (IQR 3‐5); however, this was heavily right skewed with the largest household having 17 members. Distribution of household size was similar for all infection groups (Kruskal‐Wallis *P*‐value = .2106) but different to the national urban average with fewer one‐person households in the study and more households with five or more occupants (Table S5). About 8.1% of subjects reported that at least one member of their household had ILI symptoms in the preceding week (n = 88/1083). There was no difference in the presence of household ILI between influenza‐positive and influenza‐negative groups (Kruskal‐Wallis *P*‐value = .91). Overall, 3.4% of household contacts were reported to have ILI symptoms (n = 118/3454).

## 4 DISCUSSION

Influenza surveillance in Vietnam has previously centred on hospitalised patients. This observational study was designed to investigate the incidence and associated clinical and demographic features of non‐severe influenza and influenza‐like illness in a tropical, urban primary care setting.

Influenza was present for most of the 116 weeks of the study. However, the same influenza strain did not persist but a mixture of high‐intensity peaks of single subtypes and co‐circulation of types and subtypes at variable intensities occurred. This was consistent with previous national hospital‐based surveillance which demonstrated asynchronous peaks and co‐circulation of different strains.^6^, ^8^ Our surveillance results were also consistent with data showing that the northern subtropical regions of Vietnam have a more predictable seasonality than the tropical central and southern regions.^23^ Heterogeneity in transmission patterns within tropical region is increasingly being recognised.^24^ Vietnam has one the longest and lowest “peaks” when considered nationally, and the results of this study suggest that much of this is driven by the transmission dynamics in the south of the country. This study reports higher rates of influenza positivity in ILI than are generally described globally and fewer periods where no influenza was detected.^24^, ^25^, ^26^ Attack rates measured through clinical settings are vulnerable to differences in healthcare seeking behaviour.^27^ Influenza B is typically considered to cause less severe symptoms than influenza A H3N2^28^ meaning that people are admitted less frequently and not detected through inpatient surveillance systems. This could give an impression of smaller outbreak size despite considerable community transmission.^29^, ^30^, ^31^ Influenza B was found to occur in a similar number of weeks to influenza A H3N2 but almost 100 fewer cases were detected.

Intense periods of influenza A transmission were seen in the second quarter of both 2014 and 2015 with noticeable increases after the lunar new year public holidays. Internationally, there was significant antigenic change in influenza A/H3N2 during 2014 and 2015. This manifested in a mismatch between H3N2 vaccine component and the circulating strain which resulted in reduced vaccine efficacy (<25%) and an increase in severe cases in winter 2014‐2015 in northern temperate countries.^32^, ^33^ Both periods of H3N2 activity in this study occurred at the end of the northern temperate influenza season. Genome sequencing of viruses collected during this study demonstrated modest evolution of H3N2 with most detected strains belonging to Texas/50/2012 or Switzerland/2013 antigenic groups.^34^

Primary care is the setting of choice for influenza surveillance in temperate countries,^35^ and this study demonstrates the feasibility of conducting primary care surveillance in a low middle income country across public and private providers. Recruitment at the hospital clinics was limited to daytime hours (8 am and 4 pm), whereas private clinics were able to recruit patients in the evening. All clinics had limited recruitment over the weekend period. These restrictions are likely to have impacted on the patient group which was recruited. The heterogeneity in subject demographics is important to consider when interpreting healthcare surveillance figures. In systems where patients have a relative choice regarding healthcare providers (rather than single providers like the NHS in the UK), it is important to consider alternative sources of surveillance.^31^, ^36^

Primary care surveillance relies heavily on syndromic presentations. The ability of current ILI definitions to discriminate between influenza and non‐influenza causes of upper respiratory tract infection in tropical settings has previously been called into question.^37^ The clinics in this study also participated in a trial of mobile phone reporting of ILI which demonstrated that ILI symptoms are much more consistent through the year compared to temperate countries and that peaks in ILI activity do not always correlate with peaks in influenza activity.^11^ The results of our study demonstrated that although fever, cough and rhinorrhoea were reported more commonly in those who had influenza, such symptoms also occurred in more than 70% of patients with non‐influenza ILI confirming their poor discriminating ability.

Another impact of the changing economic environment in Vietnam is the use of influenza vaccination. There is no public provision for influenza vaccination in Vietnam, and prior to 2009, the use in the general public was very rare with less than 1% of the population reporting previous vaccination.^13^ In the present study, 5% of individuals who presented to private clinics reported receiving vaccination compared to only 1.2% those who attended the hospital outpatient clinic. However, only a single individual who reported having received vaccination had a recommended indication. The increase in vaccination rates detected through this study confirms the importance of defining the appropriate vaccination strategy for tropical Vietnam.^38^ Antibiotic use prior to enrolment was high in all groups. Antibiotics can easily be purchased in pharmacies across Vietnam without prescription.^39^ Most ILI have a viral aetiology and given the short time before presentation in this study, it is unlikely that individuals have developed a secondary bacterial infection; ILI may be a major cause of inappropriate antibiotic use in this setting. Antibiotic resistance in Vietnam is an issue, as it is globally, education of medical professionals, pharmacists and the public about the appropriate use of antibiotics is urgently required.^40^

A number of limitations are present in the study design. By recruiting individuals who attended for clinical review, we will have missed those with subclinical disease. At the other end of the clinical spectrum, individuals with severe disease have been excluded. By recruiting from a variety of clinics, we attempted to recruit a representative sample of the HCMC population. Improving and broadening surveillance from hospital to the community in tropical settings is important for understanding the global spread and evolution of the virus. Mild respiratory disease is normally thought of as a single syndromic entity. Our study shows that this is an over‐simplification and that understanding the interactions between the different influenza types and subtypes, and other viral respiratory infections is critical if we are to improve prevention and management strategies for influenza and influenza‐like illness.

Influenza in southern Vietnam has complex transmission dynamics including periods of intense influenza activity of alternating types and subtypes. Broadening surveillance from hospital to the community in tropical settings is feasible and valuable for improving our understanding of the global spread and evolution of the virus.

## References

[1] Global Burden of Disease 2013 Mortality and Causes of Death Collaborators . Global, regional, and national age–sex specific all‐cause and cause‐specific mortality for 240 causes of death, 1990–2013: a systematic analysis for the global burden of disease study 2013. Lancet. 2015;385:117‐171. 25530442 10.1016/S0140-6736(14)61682-2 PMC4340604

[2] World Health Organisation . Fact sheet 211: influenza (seasonal), 2014.

[3] Ng S , Gordon A . Influenza burden and transmission in the tropics. Curr Epidemiol Rep. 2015;2:89‐100. 25938010 10.1007/s40471-015-0038-4 PMC4411750

[4] Rambaut A , Pybus OG , Nelson MI , Viboud C , Taubenberger JK , Holmes EC . The genomic and epidemiological dynamics of human influenza A virus. Nature. 2008;453:615‐619. 18418375 10.1038/nature06945 PMC2441973

[5] Russell CA , et al. The global circulation of seasonal influenza A (H3N2) viruses. Science. 2008;320:340‐346. 18420927 10.1126/science.1154137

[6] Nguyen HT , Dharan NJ , Le MT , et al. National influenza surveillance in Vietnam, 2006‐2007. Vaccine. 2009;28:398‐402. 19853073 10.1016/j.vaccine.2009.09.139

[7] Nguyen YT , Graitcer SB , Nguyen TH , et al. National surveillance for influenza and influenza‐like illness in Vietnam, 2006‐2010. Vaccine. 2013;31:4368‐4374. 23911781 10.1016/j.vaccine.2013.07.018 PMC5820022

[8] Li D , Saito R , Le MT , et al. Genetic analysis of influenza A/H3N2 and A/H1N1 viruses circulating in Vietnam from 2001 to 2006. J Clin Microbiol. 2008;46:399‐405. 17942644 10.1128/JCM.01549-07 PMC2238142

[9] Horby P , le Mai Q , Fox A , et al. The epidemiology of interpandemic and pandemic influenza in Vietnam, 2007‐2010: The Ha Nam household cohort study I. Am J Epidemiol. 2012;15:1062‐1074. 10.1093/aje/kws121 PMC3353138 22411862

[10] Nguyen HL , Saito R , Ngiem HK , et al. Epidemiology of influenza in Hanoi, Vietnam, from 2001 to 2003. J Infect. 2007;55:58‐63. 17222912 10.1016/j.jinf.2006.12.001

[11] Lam HM , Wesolowski A , Hung NT , et al. Non‐annual seasonality of influenza‐like‐illness in a tropical urban setting. bioRxiv 2017;100222 Available from: 10.1101/100222 PMC6185894 30044029

[12] Takahashi K , Suzuki M , Minh LN , et al. The incidence and aetiology of hospitalised community‐acquired pneumonia among Vietnamese adults: a prospective surveillance in Central Vietnam. BMC Infect Dis. 2013;13:296. 23815298 10.1186/1471-2334-13-296 PMC3702433

[13] Palache A , Oriol‐Mathieu V , Abelin A , Music T , Influenza Vaccine Supply task force . Seasonal influenza vaccine dose distribution in 157 countries (2004‐2011). Vaccine. 2014;32:6369‐6376. 25442403 10.1016/j.vaccine.2014.07.012

[14] Vuong CD , Hoang PM , Nguyen HL , et al. The genetic match between vaccine strains and circulating seasonal influenza A viruses in Vietnam, 2001–2009. Influenza Other Respir Viruses. 2012;7:1151‐1157. 23137010 10.1111/irv.12038 PMC4634303

[15] World Health Organisation & MOH Vietnam . Health Service Delivery Profile: Vietnam, 2012 Available from: http://www.wpro.who.int/health_services/service_delivery_profile_vietnam.pdf. Accessed November 09, 2015.

[16] European Centre for Disease Prevention and Control (ECDC) . Influenza Case Definitions. 2015. Accessed November 16, 2015.

[17] World Health Organisation . WHO Global Influenza Surveillance Network. Manual for the laboratory diagnosis and virological surveillance of influenza, 2011; Available from: http://www.who.int/influenza/gisrs_laboratory/manual_diagnosis_surveillance_influenza/en/. Accessed November 16, 2015

[18] Anders KL , Nguyen HL , Nguyen NM , et al. Epidemiology and virology of acute respiratory infections during the first year of life: a birth cohort study in Vietnam. Pediatr Infect Dis J. 2015;34:361‐370. 25674708 10.1097/INF.0000000000000643 PMC4418783

[19] World Health Organisation . Influenza Update, 2015 Available from: http://www.who.int/influenza/surveillance_monitoring/updates/latest_update_GIP_surveillance/en/. Accessed December 08, 2015

[20] Van Kerkhove MD , Broberg E , Engelhardt OG , et al. The consortium for the standardization of influenza seroepidemiology (CONSISE): a global partnership to standardize influenza seroepidemiology and develop influenza investigation protocols to inform public health policy. Influenza Other Respir Viruses. 2013;7:231‐234. 23280042 10.1111/irv.12068 PMC5779825

[21] General Statistics Office of Vietnam . Vietnam Population and housing census 2009 ‐ Age‐Sex structure and marital status of the population in Vietnam. 2009; Available from: https://www.gso.gov.vn/default_en.aspx?tabid=515&idmid=5&ItemID=11082. Accessed December 14, 2015

[22] R Core Team . R: A Language and Environment for Statistical Computing. Vienna, Austria: R Core Team; 2015.

[23] Thai PQ , Choisy M , Duong TN , et al. Seasonality of absolute humidity explains seasonality of influenza‐like illness in Vietnam. Epidemics. 2015;13:65‐73. 26616043 10.1016/j.epidem.2015.06.002

[24] Caini S , Andrade W , Badur S , et al. Temporal patterns of influenza A and B in tropical and temperate countries: what are the lessons for influenza vaccination? PLoS ONE. 2016;11:e0152310. 27031105 10.1371/journal.pone.0152310 PMC4816507

[25] Khamphaphongphane B , Ketmayoon P , Lewis HC , et al. Epidemiological and virological characteristics of seasonal and pandemic influenza in Lao PDR, 2008–2010. Influenza Other Respir Viruses. 2013;7:304‐311. 22716289 10.1111/j.1750-2659.2012.00394.x PMC5779841

[26] Lutwama JJ , Bakamutumaho B , Kayiwa JT , et al. Clinic‐ and hospital‐based sentinel influenza surveillance, Uganda 2007–2010. J Infect Dis. 2012;206(suppl 1):S87‐S93. 23169978 10.1093/infdis/jis578

[27] Brooks‐Pollock E , Tilston N , Edmunds WJ , Eames KT . Using an online survey of healthcare‐seeking behaviour to estimate the magnitude and severity of the 2009 H1N1v influenza epidemic in England. BMC Infect Dis. 2011;11:68. 21410965 10.1186/1471-2334-11-68 PMC3073914

[28] Schmier JK , Glezen WP , Kuehn CM , Ryan KJ , Oxford J . The burden of influenza B: a structured literature review. Am J Public Health. 2013;103:e43‐e51. 10.2105/AJPH.2012.301137 PMC3673513 23327249

[29] Caini S , Huang QS , Ciblak MA , et al. Epidemiological and virological characteristics of influenza B: results of the global influenza B study. Influenza Other Respir Viruses. 2015;9:3‐12. 26256290 10.1111/irv.12319 PMC4549097

[30] Cowling BJ , Ip DK , Fang VJ , et al. Modes of transmission of influenza B virus in households. PLoS ONE. 2014;9:e108850. 25268241 10.1371/journal.pone.0108850 PMC4182555

[31] Ortiz JR , Sotomayor V , Uez OC , et al. Strategy to enhance influenza surveillance worldwide. Emerg Infect Dis. 2009;15:1271‐1278. 19751590 10.3201/eid1508.081422 PMC2815958

[32] Broberg E , Snacken R , Adlhoch C , et al. Start of the 2014/15 influenza season in Europe: drifted influenza A(H3N2) viruses circulate as dominant subtype. Euro Surveill. 2015;20:21023. 25655052 10.2807/1560-7917.es2015.20.4.21023

[33] Pebody RG , Warburton F , Ellis J , et al. Low effectiveness of seasonal influenza vaccine in preventing laboratory‐confirmed influenza in primary care in the United Kingdom: 2014/15 mid‐season results. Euro Surveill. 2015;20:21025. 25677050

[34] Vy NHT , Phuong HT1 , Vinh DN1 , Boni MF . A8 The epidemiology and evolution of influenza A/H1N1 and A/H3N2 virus from 2010 to 2015, in Ho Chi Minh City, Vietnam. Virus Evol, 2017;3(Suppl 1): 10.1093/ve/vew036.007. PMC5565979 28845267

[35] Green HK , Charlett A , Moran‐Gilad J , et al. Harmonizing influenza primary‐care surveillance in the United Kingdom: piloting two methods to assess the timing and intensity of the seasonal epidemic across several general practice‐based surveillance schemes. Epidemiol Infect. 2015;143:1‐12. 25023603 10.1017/S0950268814001757 PMC9206800

[36] Ong JBS , Chen MI , Cook AR , et al. Real‐time epidemic monitoring and forecasting of H1N1‐2009 using influenza‐like illness from general practice and family doctor clinics in Singapore. PLoS ONE. 2010;5:e10036. 20418945 10.1371/journal.pone.0010036 PMC2854682

[37] Jiang L , Lee VJ , Lim WY , et al. Performance of case definitions for influenza surveillance. Euro Surveill. 2015;20:21145. 26062645 10.2807/1560-7917.es2015.20.22.21145

[38] Lambach P , Alvarez AM , Hirve S , et al. Considerations of strategies to provide influenza vaccine year round. Vaccine. 2015;33:6493‐6498. 26319745 10.1016/j.vaccine.2015.08.037 PMC8218336

[39] Nga DTT , Chuc NT , Hoa NP , et al. Antibiotic sales in rural and urban pharmacies in northern Vietnam: an observational study. BMC Pharmacol Toxicol. 2014;15:1‐10. 24555709 10.1186/2050-6511-15-6 PMC3946644

[40] Wertheim HFL , Chandna A , Vu PD , et al. Providing impetus, tools, and guidance to strengthen national capacity for antimicrobial stewardship in Viet Nam. PLoS Med. 2013;10:e1001429. 23667342 10.1371/journal.pmed.1001429 PMC3646721

