# Supplementary material for: Primary care influenza‐like illness surveillance in Ho Chi Minh City, Vietnam 2013‐2015
Source: Influenza Other Respir Viruses. 2018 Jul 7;12(5):623–31. doi: 10.1111/irv.12574 (PMC6086852; doi:10.1111/irv.12574)
Supplement: Supplementary file 1 [file IRV-12-623-s001.docx]

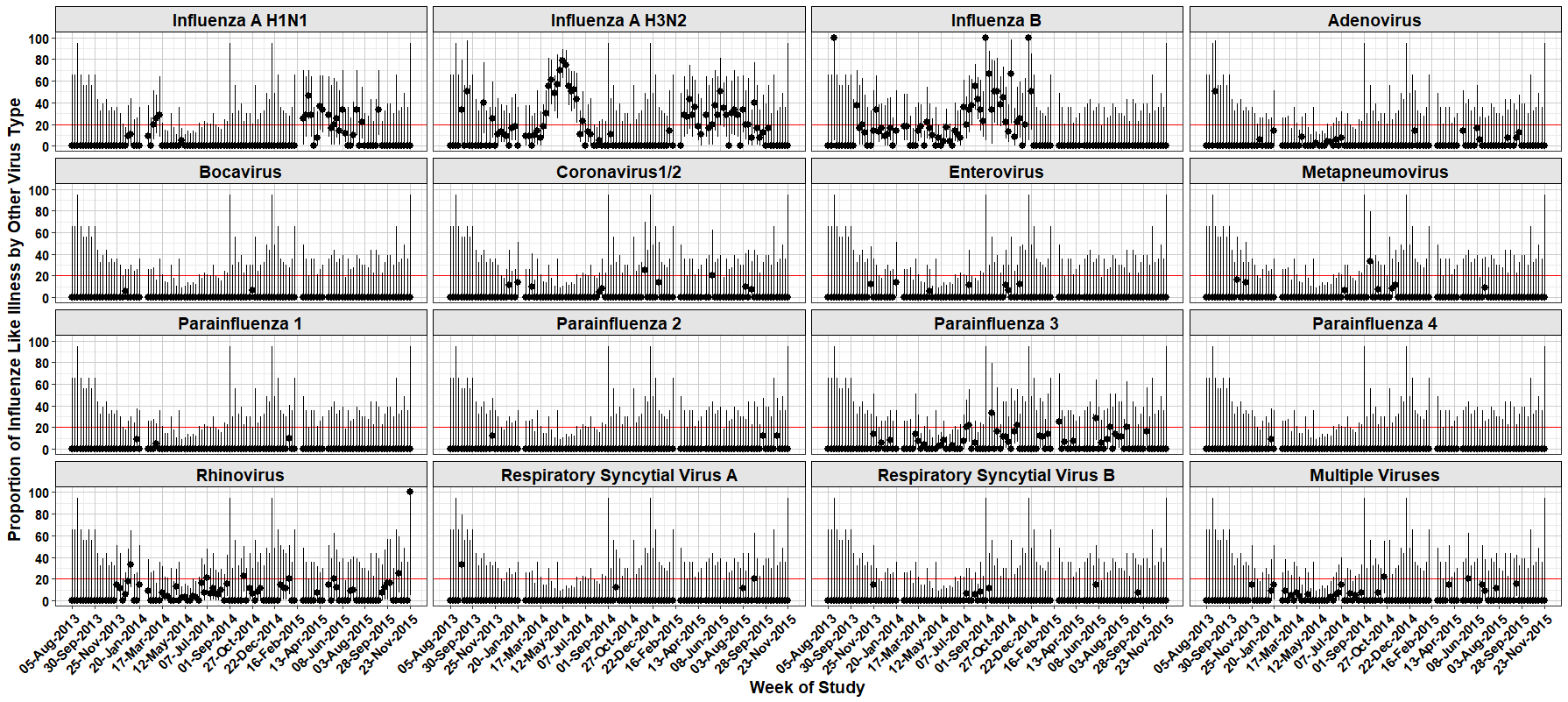


Supplementary Figure 1 Proportion of ILI caused by PCR confirmed Respiratory Viruses with Binomial 95% Confidence Intervals. Red line represents high rates of ILI positivity (20%).

| Virus | Number |
| --- | --- |
| Influenza A H1N1 | 52 (4.5) |
| Influenza A H3N2 | 258 (22.4) |
| Influenza A NT | 12 (1) |
| Influenza B | 136 (11.8) |
| Adenovirus | 17 (1.5) |
| Bocavirus | 2 (0.2) |
| Coronavirus1/2 | 10 (0.9) |
| Enterovirus | 7 (0.6) |
| Metapneumovirus | 8 (0.7) |
| Parainfluenza 1 | 3 (0.3) |
| Parainfluenza 2 | 3 (0.3) |
| Parainfluenza 3 | 44 (3.8) |
| Parainfluenza 4 | 1 (0.1) |
| Rhinovirus | 61 (5.3) |
| Respiratory Syncytial Virus A | 4 (0.3) |
| Respiratory Syncytial Virus B | 7 (0.6) |
| Multiple Viruses | 26 (2.3) |
| Negative | 501 (43.5) |

Supplementary Table 1 Influenza and Multiplex PCR Results

|  |  | Influenza A | | Influenza B | | Influenza A & B Negative | | All Study Subjects | |
| --- | --- | --- | --- | --- | --- | --- | --- | --- | --- |
|  |  | **χ^2^** | **p value** | **χ^2^** | **p value** | **χ^2^** | **p value** | **χ^2^** | **p value** |
| Vietnam National Age Distribution | **M** | 27.1 | 0.004 | 18.7 | 0.067 | 78.8 | <0.001 | 86.3 | <0.001 |
|  | **F** | 22.5 | 0.025 | 16.2 | 0.129 | 89.2 | <0.001 | 139.6 | <0.001 |
| Ho Chi Minh City Age Distribution | **M** | 37.9 | <0.001 | 38.2 | 0.001 | 66.1 | <0.001 | 136.7 | <0.001 |
|  | **F** | 20.0 | 0.047 | 16.2 | 0.131 | 68.4 | <0.001 | 90.7 | <0.001 |

Supplementary Table 2 Age and Gender Distribution by Infecting Influenza Type. Pearson's Chi Square Goodness of Fit with expected proportions in population. Symptotic p values calculated using Monte Carlo testing.

|  | Influenza A  n (%)/ med (IQR) | Influenza B  n (%)/ med (IQR) | Influenza Negative  n (%)/ med (IQR) | χ^2^ | p value |
| --- | --- | --- | --- | --- | --- |
| Age | 26.8 (19.3-36.3) | 25.4 (17.58-34.52) | 25.1 (19.7-33.6) |  |  |
| Age Category 5 to 9 | 2 (0.6) | 0 (0) | 0 (0) | 16.7662 | 0.0797 |
| 10 to 19 | 81 (25.2) | 49 (36) | 182 (26.2) |  |  |
| 20 to 44 | 196 (60.9) | 69 (50.7) | 443 (63.8) |  |  |
| 45 to 64 | 41 (12.7) | 17 (12.5) | 65 (9.4) |  |  |
| 65+ | 2 (0.6) | 1 (0.7) | 3 (0.4) |  |  |
| Gender F | 145 (45) | 60 (44.1) | 317 (45.7) | 0.1259 | 0.939 |
| M | 177 (55) | 76 (55.9) | 377 (54.3) |  |  |
| Pregnant Yes | 6 (4.2) | 2 (3.3) | 4 (1.3) | 4.6148 | 0.3291 |
| No | 137 (95.1) | 58 (96.7) | 310 (97.8) |  |  |
| DK | 1 (0.7) | 0 (0) | 3 (0.9) |  |  |
| Current Smoker Yes | 44 (13.7) | 19 (14) | 109 (15.7) | 1.482 | 0.8298 |
| No | 277 (86.3) | 117 (86) | 584 (84.1) |  |  |
| Refused | 0 (0) | 0 (0) | 1 (0.1) |  |  |
| COPD Yes | 0 (0) | 0 (0) | 2 (0.3) | 1.3222 | 0.5163 |
| No | 322 (100) | 136 (100) | 692 (99.7) |  |  |
| Asthma Yes | 5 (1.6) | 1 (0.7) | 11 (1.6) | 0.583 | 0.7471 |
| No | 317 (98.4) | 135 (99.3) | 683 (98.4) |  |  |
| Any Resp Disease Yes | 10 (3.1) | 2 (1.5) | 19 (2.7) | 0.9907 | 0.6094 |
| No | 312 (96.9) | 134 (98.5) | 675 (97.3) |  |  |
| Vaccine Indication Yes | 9 (2.8) | 3 (2.2) | 24 (3.5) | 0.7498 | 0.6873 |
| No | 313 (97.2) | 133 (97.8) | 670 (96.5) |  |  |
| Ever Received Vaccine Yes | 3 (1) | 4 (3.1) | 18 (2.6) |  |  |
| No | 309 (99) | 127 (96.9) | 667 (97.4) |  |  |
| DK | 0 (0) | 0 (0) | 0 (0) |  |  |

Supplementary Table 3 Past Medical History at Baseline

|  | Influenza A  n (%)/ med (IQR) | Influenza B  n (%)/ med (IQR) | Influenza Neg  n (%)/ med (IQR) | χ^2^ | p value |
| --- | --- | --- | --- | --- | --- |
| Occupation: At Home | 49 (15.2) | 13 (9.6) | 76 (11) | 32.2027 | 0.0208 |
| Student | 88 (27.3) | 42 (30.9) | 178 (25.6) |  |  |
| Teacher | 2 (0.6) | 4 (2.9) | 8 (1.2) |  |  |
| Office:No Public Contact | 27 (8.4) | 3 (2.2) | 40 (5.8) |  |  |
| Office:Public Contact | 30 (9.3) | 10 (7.4) | 53 (7.6) |  |  |
| Healthcare | 1 (0.3) | 0 (0) | 2 (0.3) |  |  |
| Driver | 10 (3.1) | 7 (5.1) | 22 (3.2) |  |  |
| Trader | 39 (12.1) | 16 (11.8) | 111 (16) |  |  |
| Manual Work | 59 (18.3) | 38 (27.9) | 150 (21.6) |  |  |
| Other | 17 (5.3) | 3 (2.2) | 54 (7.8) |  |  |
| Household Members | 4 (3-5) | 4 (3-5) | 4 (3-5) |  |  |
| Live Poultry Contact: never | 304 (95.6) | 130 (96.3) | 619 (91.3) | 10.0098 | 0.1242 |
| rarely | 0 (0) | 0 (0) | 1 (0.1) |  |  |
| monthly | 1 (0.3) | 0 (0) | 9 (1.3) |  |  |
| weekly | 13 (4.1) | 5 (3.7) | 49 (7.2) |  |  |
| Dead Poultry Contact:never | 262 (82.6) | 115 (85.8) | 577 (85.2) | 2.3916 | 0.8804 |
| rarely | 3 (0.9) | 1 (0.7) | 8 (1.2) |  |  |
| monthly | 17 (5.4) | 5 (3.7) | 34 (5) |  |  |
| weekly | 35 (11) | 13 (9.7) | 58 (8.6) |  |  |
| Live Pig Contact: never | 309 (97.5) | 132 (98.5) | 656 (97) | 2.9283 | 0.8178 |
| rarely | 0 (0) | 0 (0) | 1 (0.1) |  |  |
| monthly | 1 (0.3) | 1 (0.7) | 2 (0.3) |  |  |
| weekly | 7 (2.2) | 1 (0.7) | 17 (2.5) |  |  |
| Dead Pig Contact: never | 309 (97.5) | 132 (98.5) | 656 (97) | 2.9283 | 0.8178 |
| rarely | 0 (0) | 0 (0) | 1 (0.1) |  |  |
| monthly | 1 (0.3) | 1 (0.7) | 2 (0.3) |  |  |
| weekly | 7 (2.2) | 1 (0.7) | 17 (2.5) |  |  |

Supplementary Table 4 Demographic Characteristics at Baseline

|  | Influenza A (%) | Influenza B (%) | Influenza Negative (%) | National Urban Average (%) |
| --- | --- | --- | --- | --- |
| 1 person | 2.48 | 2.21 | 2.6 | 8.1 |
| 2 persons | 9.63 | 10.29 | 12.7 | 16.7 |
| 3 persons | 21.12 | 24.26 | 21.36 | 23.7 |
| 4 persons | 27.64 | 27.94 | 28.72 | 27.2 |
| 5+ persons | 39.13 | 35.29 | 34.63 | 24.3 |
| χ^2^ | 52.3 | 15.98 | 65.18 |  |
| p value | 0.0004 | 0.004 | 0.0005 |  |

Supplementary Table 5 Distribution of Household Size in Study Population and National Urban Average for Vietnam (2009). Chi Square Goodness of Fit.
